# Influence of dietary supplementation with French maritime pine bark extract (Pycnogenol^®^) on endothelial glycocalyx structures in a dynamic cell culture model

**DOI:** 10.3389/fphar.2026.1949994

**Published:** 2026-10-08

**Authors:** Maria Loka Hanna, Petra Högger

**Affiliations:** Universität Würzburg, Institut für Pharmazie und Lebensmittelchemie, Würzburg, Germany

**Keywords:** dynamic cell culture, endothelial barrier function, glycocalyx, polyphenols, Pycnogenol^®^

## Abstract

The endothelial glycocalyx (GCX) is a major contributor to endothelial cell function and vascular integrity. GCX damage has been linked to atherosclerosis promotion and other pathophysiologic states. The dietary supplement Pycnogenol®, a standardized extract from the bark of *Pinus pinaster*, has shown beneficial impact on cardiovascular and endothelial health in various clinical studies, but the underlying effect mechanism is not fully understood. The purpose of the present investigation was to explore the influence of human serum collected before and after Pycnogenol® supplementation on the properties of the endothelial GCX under basal, osmotic and inflammatory conditions. Since static cell culture poorly reproduces a physiologically relevant GCX, while controlled flow systems are technically demanding, a suitable cell culture model was to be established. A dynamic culture model using a rocking shaker was successfully implemented. Visualization of endothelial GCX structures using lectin-based staining with wheat germ agglutinin (WGA) revealed significantly increased WGA intensity (approximately 60%) under dynamic compared to static culture conditions. Under dynamic conditions, endothelial cell viability and proliferation were significantly enhanced as well as the functional responsiveness to TNF-alpha and sodium chloride exposure. When cells were dynamically incubated with serum samples obtained from healthy volunteers before and after intake of Pycnogenol®, the WGA signal intensity per cell significantly increased across all conditions. Endothelial monolayer permeability as determined with labelled dextran decreased by up to 48% after incubation with serum obtained after intake of Pycnogenol®. Moreover, the GCX shedding markers syndecan-4 and hyaluronan were significantly lower after supplementation with a significant attenuation of the response to TNF-alpha, suggesting a potential GCX protective effect by Pycnogenol® under inflammatory conditions. In summary, this study demonstrated that dynamic culture conditions promoted the formation of more pronounced glycocalyx-associated surface structures in human endothelial cells compared to static culture. In the dynamic cell culture model, application of serum samples obtained after intake of Pycnogenol® was associated with improvement of endothelial GCX properties suggesting that bioavailable and bioactive Pycnogenol® constituents and/or metabolites stabilized the endothelial GCX and improved barrier integrity.

## 1 Introduction

Cardiovascular diseases are the leading cause of death worldwide and represent a significant burden on health care systems (Vaduganathan et al., 2022). In this context, the vascular endothelium plays a key role. Metabolic and oxidative stress, such as elevated blood glucose levels, abnormal lipid profiles, or an excess of reactive oxygen species, can initiate endothelial dysfunction and subsequently vascular pathologies. Atherosclerotic vascular changes form the basis for diseases such as coronary heart disease, stroke and thrombotic events (Chistiakov et al., 2015). Given the significant contribution of impaired vascular function to the development of these diseases, the endothelial glycocalyx (GCX) has attracted considerable attention in research, as it regulates vascular permeability, mechanical signal transduction and leukocyte adhesion, thereby maintaining vascular health (Constantinescu et al., 2003; Butler et al., 2020; Moore et al., 2021). The endothelial GCX lines the vascular endothelium and forms the boundary between circulating blood and the vessel wall (Reitsma et al., 2007; Weinbaum et al., 2021). The GCX consists of endothelial-anchored glycoproteins and proteoglycans, the latter composed of core proteins such as syndecan with bound or unbound glycosaminoglycan chains, e.g., heparan sulfate or hyaluronic acid, respectively. Additionally, soluble and insoluble components were reported to be associated with this layer (Reitsma et al., 2007; Weinbaum et al., 2021; Foote et al., 2022). The dysfunction of this structure is associated with systemic infections, strokes, myocardial infarcts, and chronic diseases such as diabetes mellitus and atherosclerosis (van den Berg et al., 2006; Broekhuizen et al., 2010; Koo et al., 2013; Li et al., 2016; Martin et al., 2016; Ko et al., 2020; Gomez Toledo et al., 2025). During such conditions, GCX components such as syndecan-4 and hyaluronan are typically shed from the endothelial surface, and their elevated serum concentrations are regarded as markers of GCX degradation (Vuong et al., 2015; Diebel et al., 2019; Giuliani et al., 2024). Released GCX components such as heparan sulfate or hyaluronic acid have been suggested to exert proinflammatory functions (Kolářová et al., 2014). Shedding of central GCX components can be induced by proinflammatory cytokines such as tumor necrosis factor-alpha (TNF-alpha) (Ramnath et al., 2014). Elevated extracellular sodium concentrations, in turn, induce structural changes leading to GCX collapse through a distinct mechanism (Oberleithner et al., 2011).

There is evidence that plant-derived polyphenols contribute to cardiovascular health by improving endothelial and vascular function, possibly in part by positively influencing the formation of the GCX (Peters et al., 2015; Bharat et al., 2018; Cutler et al., 2018; Woodward et al., 2018; Iqbal et al., 2023). There is considerable interest in approaches protecting GCX structure in order to preserve vascular health (Becker et al., 2010; Sieve et al., 2018). An intensively researched polyphenolic extract is Pycnogenol® (Oliff and Blumenthal, 2010; Weichmann and Rohdewald, 2024). It is a standardized extract obtained from the bark of the French maritime pine (*Pinus pinaster* Aiton [Pinaceae]) and is characterized by a high content of oligomeric procyanidins (65%–75%) and other polyphenols (Bayer and Högger, 2024). Protective *in vitro* effects of Pycnogenol® on vascular endothelial cells have been described decades ago (Fitzpatrick et al., 1998; Virgili et al., 1998; Peng et al., 2000). In order to identify bioavailable and bioactive constituents and metabolites of Pycnogenol®, human clinical studies revealed that oligomeric procyanidins are metabolized by gut bacteria yielding the bioactive 5-(3,4-dihydroxyphenyl)-γ-valerolactone (M1) which was detected in serum, blood cells, synovial fluid and saliva samples after intake of Pycnogenol® (Bayer and Högger, 2024). Also, other polyphenols such as ferulic acid, caffeic acid and taxifolin were detectable in serum and other specimens (Grimm et al., 2006b; Mülek et al., 2017). In clinical studies, Pycnogenol® showed, e.g., anti-inflammatory and antioxidative effects as well as improvement of endothelial function parameters (Weichmann and Rohdewald, 2024). However, to the best of our knowledge, no data is currently available regarding any influence of Pycnogenol® constituents or metabolites on the GCX under inflammatory/osmotic stress conditions.

Addressing this question requires experimental models that reliably reproduce a physiologically relevant GCX. Although the importance of an intact GCX is widely recognized, its detailed analysis remains challenging (Foote et al., 2022). As a highly dynamic surface structure that is subject to a continuous balance between synthesis and degradation it is difficult to characterize and assess reliably (Lipowsky, 2005; Reitsma et al., 2007; Foote et al., 2022). Its composition and characteristics are affected by various factors, whereby shear stress from blood flow represents one of the most important stimuli. Different flow patterns determine GCX structures of varying characteristics (Maroski et al., 2011; Koo et al., 2013; Wang et al., 2020; Foote et al., 2022). In experimental studies, lectin-based visualization methods such as wheat germ agglutinin (WGA) staining are frequently used to inspect glycocalyx-associated surface structures and to assess glycocalyx-related changes in endothelial cells. However, under static conditions only an irregular and unstable GCX forms (Dane et al., 2015; Foote et al., 2022), likely because physiologically, the endothelial GCX is exposed to continuous pulsatile hemodynamic shear stress. Static *in vitro* models therefore may not accurately reflect its structural integrity and functional characteristics (Haymet et al., 2021). However, establishing controlled flow models involves considerable technical and infrastructural effort and is therefore not practicable in every experimental setting. An easily implementable method that can reproducibly stimulate the formation of a physiologically relevant GCX would be desirable. Dynamic cell culture conditions using a rocking shaker are a technically accessible option for applying mechanical stimulation to endothelial cells. However, the extent to which this form of mechanical stimulation induces a GCX structure that is both structurally and functionally relevant needs to be confirmed for individual culture settings and cell types (Sathanoori et al., 2015; Velasco et al., 2016; Ghimire et al., 2019; Warboys et al., 2019).

The aim of this study was to first establish an easily implementable dynamic HUVEC cell culture model for the formation of glycocalyx-associated surface structures. Building on this, the study sought to investigate whether Pycnogenol® supplementation influences the formation and stability of glycocalyx-associated structures under basal conditions as well as under inflammatory conditions (TNF-alpha) and osmotic stress (NaCl). Therefore, serum samples collected in the course of a clinical study (Würzburg Medical Ethics Committee approval No. 2025-47-dvh and Clinical Trial Registration No. DRKS00036277) were used in the dynamic cell culture assay in an explorative study approach. Serum samples were obtained before and after 1 week intake of 300 mg/day Pycnogenol®. It was assumed that serum samples after supplementation contain bioactive constituents and metabolites. This experimental setting has been successfully used previously (Grimm et al., 2006a; Schäfer et al., 2006).

## 2 Materials and methods

### 2.1 HUVEC cell culture and establishment of a dynamic culture model

Pooled human umbilical vein endothelial cells (HUVECs; Sigma-Aldrich, St. Louis, MO, United States) were cultured in endothelial cell growth medium (Sigma-Aldrich, Cat. No.211-500) in an incubator at 37 °C and 5% CO_2_. All culture dishes were pre-coated with 0.5% gelatin (Sigma-Aldrich). The cells (passage 2–6) were seeded into six-well plates (Sarstedt, Nümbrecht, Germany) at a density of 5 * 10^4^ per well. After 24 h of adhesion, the cells were cultured for 7 days either under static conditions or on a shaker at 23 rpm (RS-RS 6, Carl Roth, Karlsruhe, Germany), a rocking platform generating a tilting motion with a fixed amplitude of 7°. Based on the model of Zhou et al. (2010) for shear stress in rocking culture dishes, the characteristic shear stress at the well centre was estimated to be approximately 0.2 dyn/cm^2^, a magnitude consistent with shear stress levels reported in parts of the microcirculation (Cho and Cho, 2011; Park et al., 2011). This estimate is a rough approximation, as it relies on simplifying assumptions,e.g., water-like medium viscosity, idealized circular well geometry. The shaker speed was selected based on visual assessment of monolayer confluency and cell survival after 7 days of cultivation across a small number of tested speeds; 23 rpm was the highest speed at which the monolayer remained visually intact, and this speed was maintained for all further experiments. After 7 days, the wells of the static and dynamic plates were exposed to three conditions, which were always done pairwise: (I) untreated control, (II) 1-h stimulation with sodium chloride (150 mM end concentration, Sigma Aldrich) and (III) 16-h stimulation with TNF-alpha (10 ng/mL end concentration, Sigma-Aldrich). Static and dynamic conditions were always operated in parallel within the same experimental run to minimize inter-experimental variability. The experiments comprised five independent runs on different experimental days (n = 5). Each independent replicate (n) refers to one complete experimental run performed on a separate day, using freshly seeded cell cultures. The experimental setup included two series (i) the establishment of a dynamic culture model and (ii) incubation with human serum.

### 2.2 Colorimetric analysis of cell viability (CCK-8)

A CCK-8 assay (Sigma-Aldrich) was performed to evaluate cell viability. After incubations were completed, the medium in the 6-well plates was replaced with fresh medium containing 10% CCK-8 reagent and incubated for 1 h at 37 °C. Afterward, 100 µL of the supernatant was transferred to a 96-well plate (Sarstedt, Nümbrecht, Germany). Each sample was measured in duplicate; samples of different incubation conditions were randomly distributed across the plate and checked for bubbles prior to measurement. Absorbance was measured at 450 nm using an Infinite M Plex microplate reader (Tecan Group Ltd. Männedorf, Switzerland). The final result was calculated by averaging the duplicates and normalizing it according to the mean number of DAPI-positive cell nuclei in the corresponding experimental group.

### 2.3 Fluorescence microscopy and image processing

To label the GCX, the HUVECs were washed three times with PBS (Sigma-Aldrich), and fixed with 4% paraformaldehyde (Morphisto GmbH, Offenbach, Germany) for 10 min. This was followed by blocking of non-specific binding sites with 10% ROTI Immuno-Block solution (Carl Roth, Karlsruhe, Germany) for 60 min. The cells were then incubated with WGA-Alexa Fluor 488 (5 μg/mL; Invitrogen, Thermo Fisher Scientific, Waltham, MA, United States) for 60 min (in the dark, with gentle agitation). The cell nuclei were stained with DAPI (1 μg/mL; Invitrogen/Thermo Fisher Scientific) for 5 min. Each step was performed at room temperature, and three wash cycles with PBS were performed between all steps. The final measurement was performed in PBS. Image acquisition was performed using a fluorescence microscope (Observer. Z1; Zeiss, Jena, Germany) at a ×10 magnification. To minimize bias, the measurement order of the wells was randomized for each experiment. Five non-overlapping image fields were captured per well, whereby all images in this experimental series were taken with identical optimized settings. For quantitative analysis the WGA images were background-subtracted using Fiji/ImageJ (National Institutes of Health, Bethesda, MD, United States) and the fluorescence intensity of the WGA signal per field was determined. To account for differences in cell numbers, the fluorescence signal was normalized to the number of DAPI-positive nuclei in the respective field and expressed as WGA signal intensity per cell. Image fields containing fewer than 20 nuclei, saturated pixels, or visible artifacts were excluded from the analysis. For statistical analysis, the median of the five images per well was calculated and the mean value of the corresponding wells was then determined to calculate the final numeric values for the respective experiment (n).

### 2.4 Verification of the WGA binding specificity

To validate the specificity of WGA binding, a blocking experiment was performed. For this purpose, the WGA-Alexa Fluor 488 conjugate was preincubated for 30 min at room temperature with N-acetyl-D-glucosamine (final concentration 0.1 M, Sigma-Aldrich) to saturate the lectin binding sites of WGA. The subsequent staining of the cells and image acquisition were performed under identical optimized conditions as described for the standard staining protocols. Image analysis was performed analogously using ImageJ to quantify the reduction of the fluorescent signal caused by the blockade.

### 2.5 Analysis of endothelial barrier function (FITC-dextran permeability assay)

Endothelial barrier integrity was functionally assessed using a FITC-dextran permeability assay. HUVECs were cultured on Transwell inserts with a pore size of 0.4 µm in 6-well plates (Corning®, Sigma-Aldrich). After incubation with the respective stimulants, the culture medium was removed and the basolateral compartment received fresh medium, while a FITC-dextran (40 kDa, Sigma-Aldrich) solution (1 mg/mL) was added to the apical compartment. After a 1-h incubation at 37 °C, fluorescence in the lower compartment (Ex: 485 nm, Em: 520 nm) was measured. Quantification was performed using a standard curve. Permeability was calculated as the percentage of dextran passing from apical to the basolateral compartment and the permeability coefficient (P_app) was calculated according to Remenyik et al. (2022) and Vincentini et al. (2022) using the following equation:

$$P_{app}=\frac{1}{A*\;c_{0}}*\;\frac{\Delta c}{\Delta t}*\;V_{A}$$

Where A represents the membrane area, $C_{0}$ the initial concentration in the donor compartment, Δc the increase in concentration in the acceptor compartment, Δt the incubation time, and $V_{A}$ the volume of the acceptor compartment. A cell-free (blank) transwell insert was measured in parallel with each independent experiment to define the reference point for 100% diffusion.

### 2.6 Glycocalyx degradation marker determination (ELISA)

Quantitative determination of released glycocalyx degradation markers was performed employing specific enzyme-linked immunosorbent assays (ELISAs) according to the manufacturer’s instructions. Syndecan-4 concentrations were measured using a human syndecan-4 ELISA kit (Abcam, Cambridge, United Kingdom) and hyaluronan concentrations were determined using the Hyaluronan Quantikine ELISA kit (R&D Systems, Minneapolis, MN, United States). Concentrations were normalized to the corresponding DAPI-positive cell count of each sample.

### 2.7 Incubation of HUVECs with human serum

HUVECs were stimulated under shaking conditions with serum obtained from healthy volunteers (n = 6), before and after the intake of Pycnogenol®^.^ The study was reviewed and approved by the Medical Ethics Committee of the Julius-Maximilians-University Würzburg (No. 2025-47-dvh) and registered in the German Clinical Trial Register (DRKS00036277). Written informed consent was obtained from all participants prior to inclusion. Serum (10%) was added to the cell culture medium. To minimize the influence of exogenous polyphenols, the subjects followed a polyphenol-restricted diet for 48 h. Following this phase the first blood sample was collected (baseline; “before intake”). This was followed by 1 week of supplementation with Pycnogenol® (300 mg/day), after which the second blood sample (“after intake”) was obtained. The serum samples from all donors were pooled for each condition (before vs. after intake) to focus on general effects instead of donor-specific reactions. While the WGA staining, permeability assay and the glycocalyx degradation marker determination were performed with n = 5 independent experiments, the evaluation of cell viability (CCK-8) and the WGA-blocking were performed with n = 3 independent experiments due to the limited availability of human serum.

### 2.8 Statistical analysis

Statistical analyses were performed using GraphPad Prism version 11.0.0 for Windows (GraphPad Software, LA Jolla, CA, United States). Tests were based on the distribution of the data which was assessed using the Shapiro-Wilk test. For analysis of WGA staining, the CCK-8 assay, the WGA-blockade, the permeability assay and the ELISA results, a two-way ANOVA followed by a Tukey *post hoc* test was applied. Relative changes compared with the respective conditions were analyzed using a one-sample t-test. A p-value of p < 0.05 was defined as statistically significant.

## 3 Results

### 3.1 Establishment of a dynamic culture model

#### 3.1.1 Analysis of cell viability assay (CCK-8)

After the incubation with the respective stimulants, a confluent monolayer was formed. Visually, no morphological differences were observed between the static and dynamic culture conditions. However, static conditions tended to result in a higher number of DAPI-positive nuclei per image compared to dynamic conditions (211 ± 10 versus 155 ± 10 nuclei per field; Wilcoxon test: p-value = 0.0625). To rule out cytotoxic effects as a confounding factor and to assess cell viability, a CCK-8 assay was performed. Dynamic cultivation resulted in a significantly higher normalized CCK-8 signal across all treatment groups compared to the corresponding static controls (Figure 1). This relative increase was most pronounced under control conditions (53.55% ± 11.13%, p-value = 0.0086) and NaCl treatment (47.46% ± 13.30%, p-value = 0.0243). The increase under TNF-alpha stimulation was less pronounced with 22.21% ± 4.47% but still significant (p-value = 0.0077). The corresponding absolute values for all conditions, along with the associated standard errors (SEMs) and p-values, are summarized in Table 1. Individual values for all experiments are provided in the Supplementary Material (Supplementary Table S1).

**FIGURE 1:**
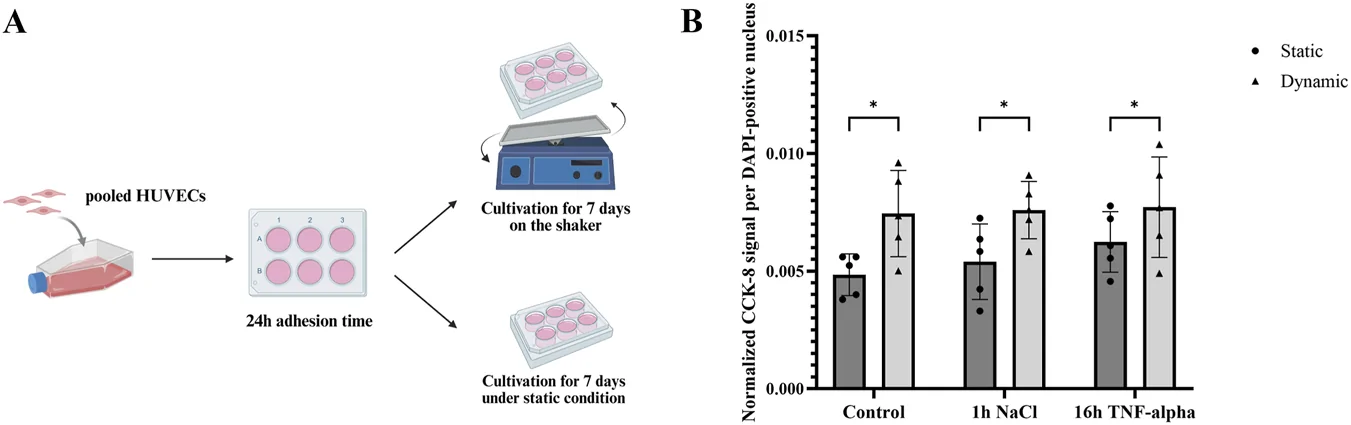
Establishment of the dynamic culture model and CCK-8 assay. **(A)** Schematic overview of the experimental setup: HUVECs were seeded and allowed to adhere for 24 h, followed by cultivation for 7 days either on a rocking shaker (dynamic condition) or under static conditions without applying any agitation to the culture dishes. **(B)** CCK-8 assay of HUVECs cultured under static and dynamic cell culture conditions. CCK-8 absorbance was normalized to the number of DAPI-positive nuclei (representative DAPI staining is shown as part of the WGA/DAPI panels in Figure 3). Data are presented as mean with SEM of five independent experiments (n = 5). Statistical significance was assessed using two-way ANOVA followed by Tukey’s *post hoc* test. Panel **(A)** Created in BioRender. Högger, P. (2026) https://BioRender.com/zqqxvvf.

**TABLE 1:** Normalized CCK-8 absorbance values under static and dynamic culture conditions.

Condition	Static	Dynamic	p-value^†^
Control	0.004844 ± 0.000396	0.007446 ± 0.000821	0.0110
1 h NaCl	0.005393 ± 0.000718	0.007594 ± 0.000545	0.0127
16 h TNF-alpha	0.006235 ± 0.000575*	0.007716 ± 0.000956	0.0187

Data are presented as mean ± SEM, from five independent experiments (n = 5).

^†^ Comparison between static and dynamic conditions within the respective treatment group (two-way ANOVA, followed by Tukey *post hoc* test).

* p < 0.05 compared with the respective untreated control (two-way ANOVA, followed by Tukey *post hoc* test).

#### 3.1.2 WGA signal intensity per cell

Compared with static culture conditions, dynamic cultivation significantly increased the WGA signal intensity per cell (24,909 ± 570 vs. 39,528 ± 2,466; p-value = 0.0042; Figure 2A). Representative images are shown in Figures 3A,B. Cells exposed to NaCl (+25.45 ± 11.33%) or TNF-alpha (+11.80 ± 6.34%) also showed an increase in the WGA signal intensity per cell under dynamic as compared to static culture conditions. Exposure to NaCl or TNF-alpha did not significantly affect the WGA signal intensity per cell under static controls compared to its control (−2.39% ± 5.83% and −3.85% ± 6.50%). Under dynamic culture, NaCl and TNF-alpha both reduced significantly the WGA signal intensity per cell relative to the untreated dynamic control (−23.66% ± 4.06% and −32.40% ± 3.96%; p-value = 0.0043 and 0.0012). The corresponding absolute values for all conditions, as well as the associated SEMs and p-values, are listed in Table 2. Individual values for all experiments are provided in the Supplementary Material (Supplementary Table S2).

**FIGURE 2:**
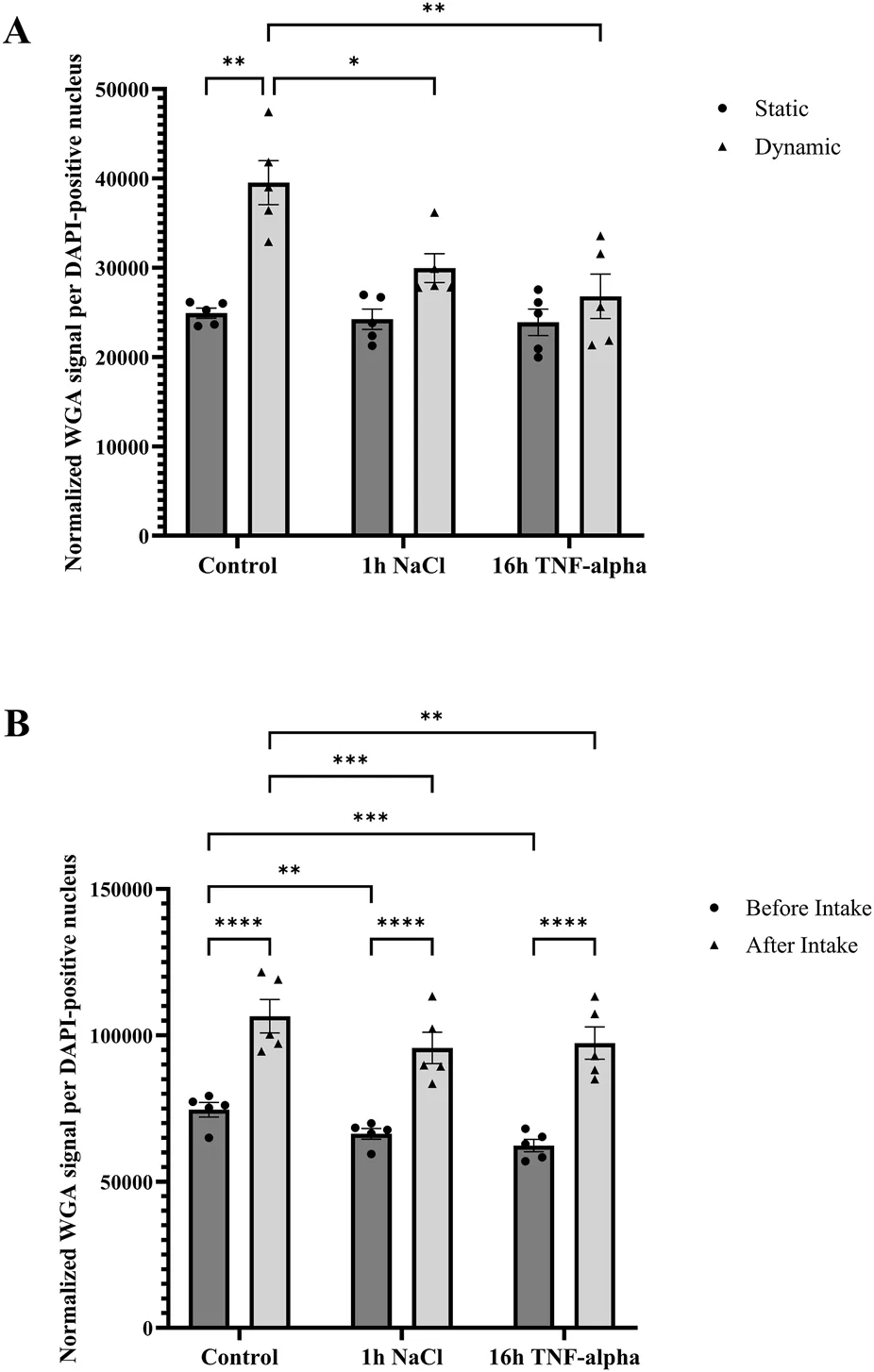
Quantification of WGA fluorescence intensity per cell: **(A)** Comparison of static and dynamic culture conditions after 7 days of cultivation (representative images shown in Figures 3A,B), **(B)** Dynamic cell culture conditions when cells were cultured with serum samples from human volunteers obtained before and after supplementation with Pycnogenol® (representative images shown in Figures 3C,D). Data are presented as mean with SEM from five independent experiments (n = 5; serum from n = 6 donors pooled per condition). Statistical significance was assessed using two-way ANOVA followed by Tukey’s *post hoc* test.

**FIGURE 3:**
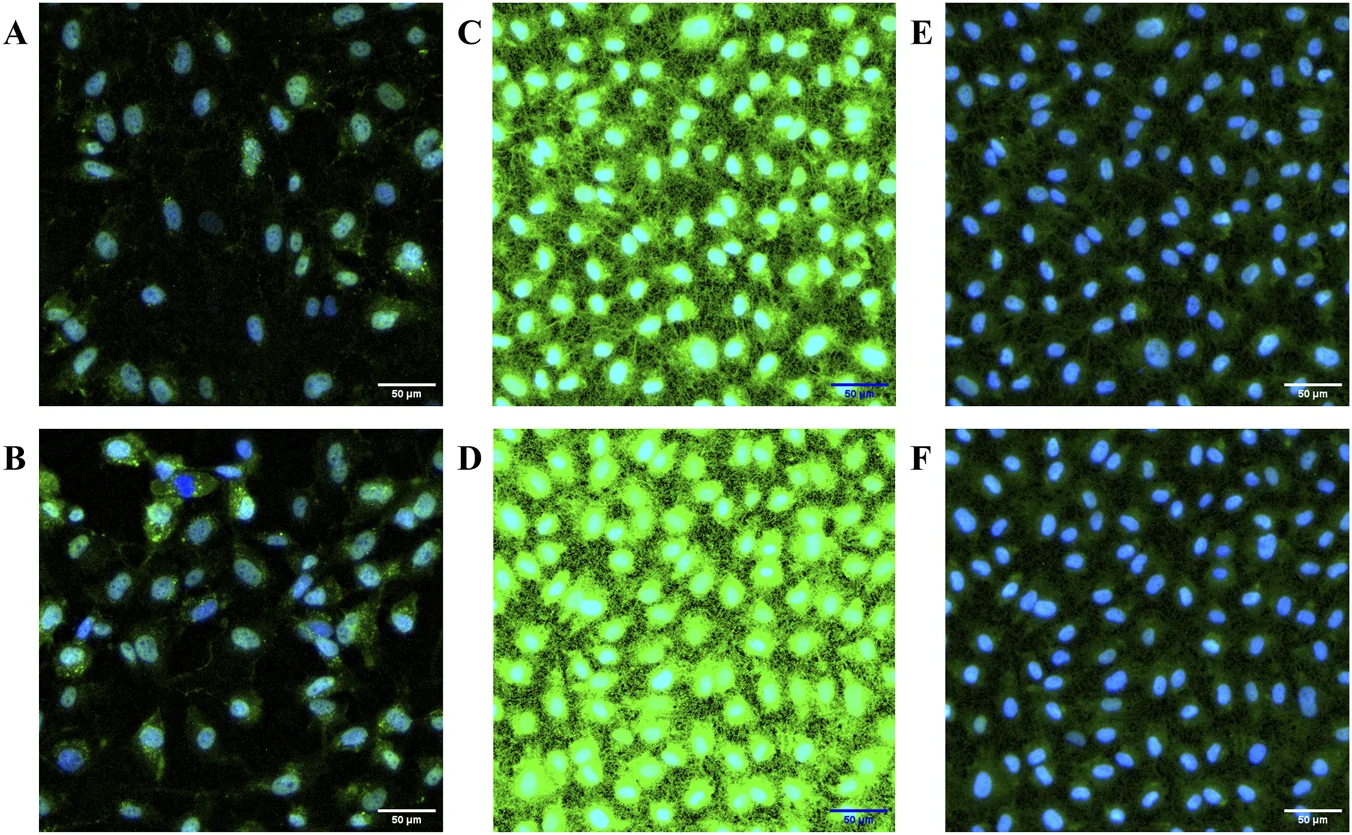
Representative fluorescence images of HUVECs stained with WGA-Alexa Fluor 488 (green) and DAPI (blue): **(A–D)** All images were acquired and processed using identical settings to ensure comparable representation of differences in WGA intensity across conditions. **(A)** Static culture **(B)**, Dynamic culture **(C)**, Dynamic culture with human serum before supplementation, **(D)** Dynamic culture with human serum after supplementation. **(E,F)** Contrast-enhanced sections of the images shown in **(C,D)** presented for qualitative visualization only. These panels were not used for quantification.

**TABLE 2:** WGA fluorescence intensity per cell.

Condition	Static	Dynamic	p-value^†^
Control	24,909 ± 570	39,528 ± 2,466	0.0042
1 h NaCl	24,221 ± 1,139	29,940 ± 1614^*^	0.0813
16 h TNF-alpha	23,886 ± 1,476	26,793 ± 2492^**^	0.1464

​	Before intake	After intake	​
Control	74,581 ± 2,488	106,525 ± 5,723	<0.0001
1 h NaCl	66,377 ± 1831^**^	95,664 ± 5377^***^	<0.0001
16 h TNF-alpha	62,327 ± 2091^***^	97,327 ± 5521^**^	<0.0001

Data are presented as mean ± SEM from five independent experiments (n = 5).

^†^ Comparison between conditions within the respective treatment group (two-way ANOVA followed by Tukey *post hoc* test).

* p < 0.05, **p < 0.01, ***p < 0.001 compared with respective untreated control (two-way ANOVA followed by Tukey *post hoc* test).

#### 3.1.3 Verification of binding specificity

Preincubation of the WGA conjugate with N-acetyl-D-glucosamine resulted in a significant reduction in the fluorescence signal of 80.69% ± 0.88% (p-value = < 0.0001) under all experimental conditions compared to the respective unblocked controls. The corresponding absolute values for all conditions, along with the associated SEMs are provided in the Supplementary Material (Supplementary Table S3).

#### 3.1.4 Analysis of endothelial barrier function (FITC-dextran permeability assay)

Endothelial barrier function was assessed using a FITC-dextran permeability assay. Neither the FITC-dextran permeability (%) nor the apparent permeability coefficient differed significantly between static and dynamic culture conditions. Detailed results are provided in the Supplementary Table S4.

#### 3.1.5 Determination of glycocalyx degradation markers (ELISA)

Quantification of glycocalyx-associated markers revealed higher concentrations of hyaluronan and syndecan-4 under dynamic culture conditions compared with static culture conditions. Hyaluronan concentrations were significantly increased in the untreated and NaCl-treated groups. Although hyaluronan concentrations were also numerically higher in the TNF-alpha-treated group under dynamic conditions, this difference did not reach statistical significance. Similarly, syndecan-4 concentrations were significantly higher under dynamic culture conditions in the untreated and TNF-alpha treated groups. No significant differences were observed between NaCl-treated static and dynamic cultures. The corresponding absolute values for all conditions, as well as the associated SEMs and p-values, are listed in Table 3. Detailed results are provided in Supplementary Table S5.

**TABLE 3:** Concentrations of syndecan-4 and hyaluronan.

Condition	Static (pg/10^5^ cells)	Dynamic (pg/10^5^ cells)	p-value^†^
Syndecan-4			
Control	114.6 ± 5.1	129.8 ± 2.5	0.0217
1 h NaCl	117.8 ± 4.7	127.3 ± 1.5	0.1416
16 h TNF-alpha	124.0 ± 4.6^*^	136.3 ± 2.0	0.0280
Hyaluronan			
Control	47.4 ± 2.0	60.6 ± 4.5	0.0135
1 h NaCl	49.2 ± 3.2	58.43 ± 2.8	0.0226
16 h TNF-alpha	51.7 ± 2.9	64.08 ± 4.4	0.0505

​	Before intake (pg/10^5^ cells)	After intake (pg/10^5^ cells)	​
Syndecan-4			
Control	39.11 ± 3.64	27.11 ± 0.58	0.0201
1 h NaCl	36.71 ± 3.54	25.45 ± 1.02	0.0292
16 h TNF-alpha	46.47 ± 4.66^*^	29.73 ± 0.56^*^	0.0183
Hyaluronan			
Control	70.66 ± 4.53	52.48 ± 3.99	0.0019
1 h NaCl	76.58 ± 6.02	55.24 ± 3.53^*^	0.0064
16 h TNF-alpha	85.52 ± 4.62^**^	58.52 ± 2.67	0.0014

Data are presented as mean ± SEM from five independent experiments (n = 5).

^†^ Comparison between conditions within the respective treatment group (two-way ANOVA followed by Tukey *post hoc* test).

* p < 0.05, **p < 0.01 compared with respective untreated control (two-way ANOVA followed by Tukey *post hoc* test).

### 3.2 Incubation of HUVECs with human serum samples

#### 3.2.1 Analysis of cell viability assay (CCK-8)

Incubation with the pooled human serum samples (before/after intake) did not negatively affect cell morphology over the entire 7-day period; a stable and confluent monolayer was formed in all groups. Counting of DAPI-positive nuclei revealed nearly identical cell density between the two serum groups (339 ± 32 vs. 336 ± 30 nuclei per field; paired t-test, p-value: 0.8385). Consistent with this, the result of the CCK-8 assay showed that samples obtained after supplementation with Pycnogenol® did not significantly affect the viability of the cells. Also, within a serum group, the stimulants (NaCl and TNF-alpha) had no significant effect on cell viability compared with the respective control condition. The corresponding absolute values for all conditions, as well as the associated SEMs and p-values, are provided in the Supplementary Material (Supplementary Table S6).

#### 3.2.2 WGA signal intensity per cell

Quantitative analysis showed higher WGA signal intensity per cell in all conditions after supplementation compared with the “before intake” serum group (Figure 2B). Under basal conditions, the WGA signal intensity per cell was 42.85% ± 5.93% higher in the “after intake” serum group compared with the “before intake” serum group (p-value = 0.0019). Representative fluorescence images cultured with serum are shown in Figures 3C–F. Higher WGA signal intensity per cell was observed under NaCl incubation (43.87% ± 5.44%, p-value = 0.0013) and TNF-alpha stimulation (56.72% ± 9.76%, p-value = 0.0044) when the cells were exposed to the serum samples obtained after intake as compared to before intake of Pycnogenol®. In the respective serum groups (“before/after intake”), different relative changes were observed as response to NaCl and TNF-alpha. In the “before intake” group, exposure to NaCl led to a relative reduction of the WGA signal intensity per cell of 10.91% ± 0.92% (p-value = 0.0003) and stimulation with TNF-alpha resulted in a relative reduction of 16.28% ± 2.45% (p-value = 0.0027). In the “after intake” group, NaCl induced a relative reduction of the WGA signal intensity per cell of 10.16% ± 1.89% (p-value = 0.0057), while the signal loss under TNF-alpha stimulation was only 8.60% ± 2.12% (p-value = 0.0153). The corresponding absolute values for all conditions, along with the associated SEMs and p-values, are summarized in Table 2. Individual values for all experiments are provided in the Supplementary Material (Supplementary Table S2).

#### 3.2.3 Verification of binding specificity

The specificity of the WGA signal intensity per cell in the presence of serum was confirmed by a blocking experiment with N-acetyl-D-glucosamine. Preincubation resulted in an average reduction in fluorescence intensity of 80.92% ± 0.88% (p-value = < 0.0001) across all experimental conditions. The corresponding absolute values for all conditions, along with the associated SEMs are provided in the Supplementary Material (Supplementary Table S3).

#### 3.2.4 Analysis of endothelial barrier function (FITC-dextran permeability assay)

Analysis of the endothelial permeability demonstrated a significantly lower permeability in all experimental conditions when serum collected after supplementation was used, compared with serum collected before supplementation (Figure 4). Relative to the corresponding “before intake” groups, permeability decreased by 43.02% ± 1.10% (p-value <0.0001) under control condition, by 42.59% ± 0.76% (p-value <0.0001) after NaCl exposure and by 49.25% ± 1.02% (p-value <0.0001) after TNF-alpha stimulation. Similar results were obtained for both FITC-dextran permeability (%) and P_app (Table 4). Within the respective serum groups, NaCl exposure significantly increased endothelial permeability compared with the corresponding untreated controls. The magnitude of this increase was comparable between the “before intake” and “after intake” serum groups and amounted to 20.85% ± 0.74% (before intake) and 21.81% ± 0.77% (after) (both p-value <0.0001). TNF-alpha stimulation also significantly increased endothelial permeability in both serum groups. However, the magnitude of this effect differed between the two conditions. In the “before intake” serum group, permeability increased by 42.77% ± 1.85% (p-value <0.0001) compared to control, whereas the increase was only 27.11% ± 1.27% (p-value <0.0001) in the “after intake” serum group. The corresponding absolute values for all conditions, along with the associated SEMs and p-values, are summarized in Table 4. Individual values for all experiments are provided in the Supplementary Material (Supplementary Table S7).

**FIGURE 4:**
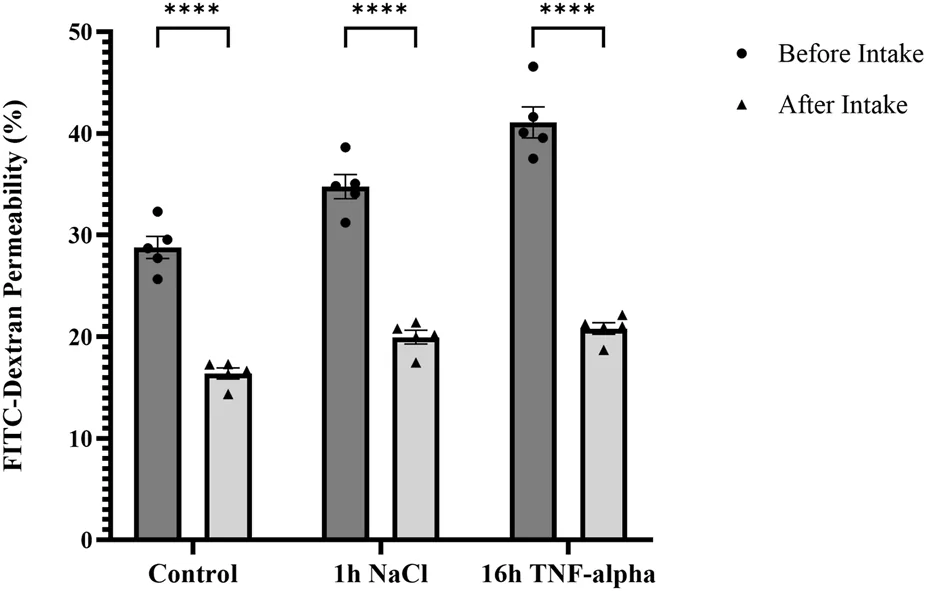
FITC-dextran permeability assay under dynamic cell culture conditions when cells were cultured with serum samples from human volunteers obtained before and after supplementation with Pycnogenol®. Endothelial permeability is expressed as FITC-dextran permeability (%). Data are presented as mean with SEM from five independent experiments (n = 5; serum from n = 6 donors pooled per condition). Statistical significance was assessed using two-way ANOVA followed by Tukey’s *post hoc* test.

**TABLE 4:** Results of FITC-dextran permeability (%) and P_app (*10^−6^ cm/s) under dynamic conditions with serum.

Condition	Before intake	After intake	p-value^†^
FITC-dextran permeability (%)			
Control	28.78 ± 1.09	16.38 ± 0.54	<0.0001
1 h NaCl	34.76 ± 1.19^****^	19.95 ± 0.67^****^	<0.0001
16 h TNF-alpha	41.07 ± 1.53^***^	20.80 ± 0.57^****^	<0.0001
P_app (*10^−6^ cm/s)			
Control	1.77 ± 0.33	1.01 ± 0.20	<0.0001
1 h NaCl	2.13 ± 0.38^****^	1.23 ± 0.23^***^	<0.0001
16 h TNF-alpha	2.50 ± 0.42^****^	1.28 ± 0.23^****^	<0.0001

Data are presented as mean ± SEM from five independent experiments (n = 5). The mean permeability coefficient (P_app) of a cell-free (blank) transwell insert, measured in parallel with each independent experiment, was 5.82 ± 1.50 ×10^−6^ cm/s (n = 5).

^†^ Comparison between before and after intake conditions within the respective treatment group (two-way ANOVA, followed by Tukey *post hoc* test).

* p < 0.05, **p < 0.01, ***p < 0.001, ****p < 0.0001 compared with respective untreated control (two-way ANOVA followed by Tukey *post hoc* test).

#### 3.2.5 Determination of glycocalyx degradation markers (ELISA)

Quantitative analysis of glycocalyx-associated markers revealed significantly lower concentrations of both syndecan-4 and hyaluronan across all three treatment conditions, when cells were exposed to serum collected after supplementation compared with serum collected before supplementation (Figures 5A,B). Compared to the cells exposed to serum without supplementation, syndecan-4 concentrations were reduced by 28.61% ± 5.57% (p-value = 0.0068) under control conditions, by 28.53% ± 6.15% (p-value = 0.0097) after NaCl and by 33.77% ± 5.71% (p-value = 0.0041**)** after TNF-alpha stimulation. Similar reductions were observed for hyaluronan, which were 25.65% ± 3.14% (p-value = 0.0012), 27.10% ± 3.97% (p-value = 0.0024), and 31.23% ± 2.96% (p-value = 0.0005), under the respective conditions. Within the “before intake” serum group, TNF-alpha stimulation significantly increased both syndecan-4 and hyaluronan concentrations relative to the untreated controls. After supplementation, the TNF-alpha-associated increases were still present, but smaller. The corresponding absolute values for all conditions, along with the associated SEMs and p-values, are summarized in Table 3. Individual values for all experiments are provided in the Supplementary Material (Supplementary Table S8).

**FIGURE 5:**
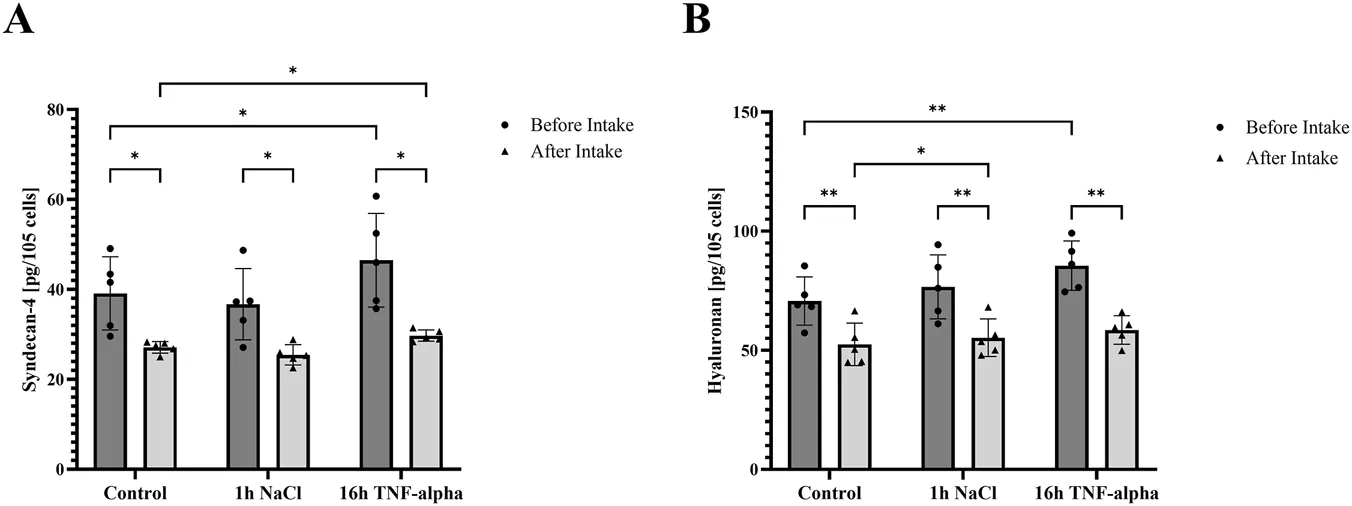
Quantification of glycocalyx-associated markers under dynamic cell culture conditions when cells were cultured with serum samples from human volunteers obtained before and after supplementation with Pycnogenol®: **(A)** Syndecan-4 concentrations **(B)** Hyaluronan concentrations. Data are presented as mean ± SEM from five independent experiments (n = 5; serum from n = 6 donors pooled per condition). Statistical significance was assessed using two-way ANOVA followed by Tukey’s *post hoc* test.

## 4 Discussion

The aim of the present study was to establish a cell culture setting that produced a more pronounced glycocalyx-associated surface structure on endothelial cells compared to regular culture conditions. Based on this, the effect of Pycnogenol® supplementation on the GCX structure was investigated under conditions simulating inflammatory (TNF-alpha) or osmotic stress (NaCl).

A key finding of this study was an almost 60% increase in the WGA signal intensity per cell under dynamic culture conditions compared to static culture. Since WGA binds specifically to sialic acid and N-acetyl-D-glucosamine, which are essential components of glycoproteins and proteoglycans (Li and Wang, 2018), the increased signal was consistent with enhanced formation of endothelial GCX structures. The marked reduction in the WGA signal intensity per cell following preincubation with N-acetyl-D-glucosamine confirmed the specificity of the staining for glycosylated surface structures and thus supported the conclusion that the observed differences were indeed associated with the GCX. This is consistent with previous studies showing that shear stress promotes GCX development (Gouverneur et al., 2006) and endothelial cells maintained under standard static culture conditions fail to assemble a hydrodynamically relevant GCX (Potter and Damiano, 2008; Potter et al., 2009). Earlier studies characterized an orbital shaker model as an accessible *in vitro* approach that exposes endothelial cells to shear stress (Velasco et al., 2016; Warboys et al., 2019). In line with this, the horizontal shaker model used in the present study promoted the formation of glycocalyx-associated structures and enhanced endothelial responsiveness, supporting its suitability.

Under dynamic cell culture conditions, both NaCl and TNF-alpha reduced the WGA signal compared to control, whereas no such effect was observed under static conditions, suggesting that glycocalyx-associated surface structures were not sufficiently developed to be impaired by either stimulus. An intact GCX has been shown to play an important role in mechanosensitive endothelial responses, as selective removal of individual GCX components was sufficient to block shear-induced signaling (Pahakis et al., 2007). The decrease of the WGA signal by TNF-alpha and NaCl is consistent with their known damaging effects on the glycocalyx: TNF-alpha can promote the shedding of central glycocalyx components, while elevated extracellular sodium concentrations can induce structural changes up to collapse of the glycocalyx (Oberleithner et al., 2011; Ramnath et al., 2014).

Under all dynamic culture conditions, an increased normalized viability (CCK8) signal per cell was observed compared to static culture. The CCK-8 assay is widely used to evaluate the cell viability and proliferation of cells (Heath et al., 2010; Zuo et al., 2026). Mechanical stimuli such as shear stress activate various signaling pathways in endothelial cells and, for example, promote ATP release and mitochondrial respiration (Milner et al., 1990; Kim et al., 2014; Fang et al., 2019). Therefore, the increased CCK-8-signal under dynamic culture conditions was consistent with a mechanically induced stimulation of endothelial cell function and proliferation. Beyond the comparison between static and dynamic culture conditions, the CCK-8 signal was not impaired by exposure of HUVECs to NaCl or TNF-alpha, suggesting unaffected cell viability.

Under dynamic culture conditions, DAPI staining revealed a lower number of cell nuclei per image field compared to static culture, most likely reflecting a mechanically induced detachment of individual cells. This is in line with reports that an abrupt change in mechanical conditions can promote cytoskeletal reorganization and endothelial cell detachment (Inglebert et al., 2020). Despite the lower cell count under dynamic culture conditions, the normalized CCK-8 signal increased compared to static culture, suggesting that the remaining adherent cells responded to the mechanical stimulus with increased functional activity of the endothelial monolayer.

The glycocalyx-related changes under dynamic culture conditions were not reflected in a change in FITC-dextran permeability. Therefore, although the GCX may have undergone structural changes under the selected experimental conditions, permeability was not measurably affected. This notion is supported by the subsequent experiments supplementing human serum samples to the cell culture. Hereby, the same experimental setup revealed significant and reproducible differences in endothelial permeability between the groups, confirming the general sensitivity and suitability of the permeability assay.

Under dynamic culture conditions, elevated syndecan-4 and hyaluronan concentrations were determined in the supernatant. The GCX represents a dynamic structure in which a continuous equilibrium exists between biosynthesis and shedding of its components, regulated by fluid shear stress (Reitsma et al., 2007). In this context, Gouverneur et al. demonstrated that shear stress simultaneously increases hyaluronan content within the GCX and hyaluronan concentrations in the supernatant (Gouverneur et al., 2006), indicating that elevated extracellular marker levels reflect increased GCX turnover rather than monolayer degradation. In the present study, the functional activity (CCK8), permeability (FITC-dextran) and morphology of the cell monolayer did not indicate cell damage, which argues against a relevant decrease of cell viability. The elevated syndecan-4 and hyaluronan concentrations observed under dynamic culture conditions may likewise reflect increased turnover of glycocalyx-associated components rather than GCX loss. Heterogeneity was observed between the two markers: the increase from static to dynamic culture did not reach statistical significance for hyaluronan following TNF-alpha stimulation, and for syndecan-4 following NaCl-stimulation. This might be due to differences in the regulation and release kinetics of these two GCX markers, since syndecans are membrane-bound core proteoglycans, whereas hyaluronan represents a structurally distinct glycosaminoglycan which is indirectly bound to the GCX via its receptor CD44 (Reitsma et al., 2007). Yet, no orthogonal structural or intracellular measures to distinguish increased turnover from net degradation were conducted in the present study, which represents a limitation of the marker-interpretation.

Since a more pronounced GCX was formed in the dynamic cell culture and serum sample volumes were limited, all subsequent experiments with serum samples were exclusively performed under dynamic conditions, in a test system that was presumably functionally closer to the physiological vascular endothelium. Generally, the addition of serum to the culture medium would be expected to help preserve the GCX structure, for example by the supplementation of additional albumin. Albumin accounts for approximately 50% of the total protein content in the plasma of healthy individuals and has been shown to support the structural integrity of the GCX (Becker et al., 2010; Aldecoa et al., 2020).

Significantly enhanced formation of endothelial GCX structures by roughly 43% based on the WGA signal intensity per cell was observed when cells were incubated with serum samples obtained after intake of Pycnogenol® as compared to samples before intake. This is consistent with the possibility that, following supplementation, Pycnogenol® constituents and/or metabolites contributed to the formation or stabilization of endothelial glycocalyx-associated structures. As expected, under osmotic stress (NaCl) and proinflammatory stimulation (TNF-alpha) the GCX structures were reduced, but the WGA signal remained to be significantly higher after incubation with serum after intake of Pycnogenol® compared to serum before intake. The dietary supplementation did not affect cell viability under any of the experimental conditions as assessed by the CCK8 assay. Improvement of GCX structures has been reported for other plant-derived compounds as well. Surface glycosaminoglycans of human aortic endothelial cells were restored when they were incubated with a mixture of phenolic blueberry metabolites. Furthermore, a reduced expression of inflammatory markers, e.g., of the adhesion molecule VCAM1 was observed (Cutler et al., 2018). Single polyphenols such as resveratrol, quercetin or epicatechin at concentrations of 10 μg/mL induced swelling of the endothelial GCX and reduced membrane stiffness (Peters et al., 2015). An increased GCX volume and reduced GCX stiffness was also seen in endothelial cells being incubated with a procyanidin-containing hawthorn extract at concentrations of 10 μg/mL (Peters et al., 2012). Therefore, our observations are consistent with previous reports. A notable aspect of the present study is that effects were detected with serum samples containing the full spectrum of physiologically occurring and bioavailable constituents and metabolites after intake of Pycnogenol®. These serum samples, representing 10% of the cell culture medium, probably contained concentrations between 0.004–0.1 μg/mL of individual polyphenolic compounds as determined in previous pharmacokinetic investigations with a single dose of 300 mg Pycnogenol® (Bayer and Högger, 2024).

The endothelial permeability, as assessed with FITC-dextran, significantly decreased when cells were incubated with serum samples obtained after intake of Pycnogenol® as compared to samples before intake, might indicate a possible contribution of Pycnogenol® constituents and/or metabolites to enhanced integrity of the endothelial barrier. Both osmotic stress and proinflammatory stimulation increased the FITC-dextran permeability. An increased dextran flux after TNF-alpha stimulation is consistent with previous reports (Henry and Duling, 2000; Kunimura et al., 2021). Elevated sodium concentrations have been shown to damage the endothelial glycocalyx (Oberleithner et al., 2011), which is an important determinant of endothelial barrier function and whose impairment is associated with increased permeability (Butler et al., 2020). In the present study, serum samples obtained after the intake of Pycnogenol® significantly improved the endothelial barrier function also in the presence of NaCl and TNF-alpha. Notably, the increase in permeability induced by TNF-alpha was markedly reduced when cells were exposed to serum after intake of Pycnogenol®, indicating an attenuation of the TNF-alpha response. The NaCl-induced increase in permeability remained unchanged after cells were incubated with serum obtained after intake of Pycnogenol®. An improved endothelial barrier resistance to sodium was previously also observed after incubation of cells with 10 μg/mL of a hawthorn extract. The authors suggested that this effect contributed to the vasoprotective action of the extract (Peters et al., 2012). A TNF-alpha-induced vascular endothelial barrier dysfunction was significantly improved after addition of 300 µM of a non-phenolic, sulfur-containing compound which is present in aged garlic extracts. The protection of junctional proteins on the cell membrane was identified as the underlying mechanism of the compound activity (Kunimura et al., 2021).

Shedding of syndecan-4 and hyaluronan indicates damage to the GCX, with TNF-alpha promoting the enzymatic shedding of GCX components and elevated sodium concentrations causing collapse of the GCX (Oberleithner et al., 2011; Ramnath et al., 2014; Vuong et al., 2015; Diebel et al., 2019; Giuliani et al., 2024). Following Pycnogenol® supplementation, lower concentrations were measured both for syndecan-4 and hyaluronan under all experimental conditions. In contrast to the comparison between static and dynamic culture conditions, where increased marker concentrations were associated with increased metabolic activity and were therefore interpreted as reflecting enhanced endothelial turnover of GCX components rather than damage. The comparison between the two serum groups allowed for a different interpretation: since metabolic activity did not differ between serum groups, the lower concentrations for syndecan-4 and hyaluronan observed following supplementation more likely indicated a stabilizing effect on glycocalyx-associated structures rather than reduced turnover. Upon exposure to NaCl, no significant increase of syndecan-4 and hyaluronan concentrations was recorded for cells incubated with serum samples obtained before intake of Pycnogenol®. This would be consistent with the fact that osmotic stress primarily collapses the GCX (Oberleithner et al., 2011). However, still a significant decrease of release of syndecan-4 and hyaluronan into cell culture supernatant was seen when osmotically stressed cells were exposed to serum samples collected after as compared to before intake of Pycnogenol®. While TNF-alpha significantly increased shedding of syndecan-4 and hyaluronan of HUVECs incubated with serum collected prior to intake of Pycnogenol®, this effect was less pronounced when cells were exposed to serum samples after intake of the dietary supplement. These findings suggest that constituents and/or metabolites of Pycnogenol® may have stabilized glycocalyx-associated structures, e.g., by decreasing shedding activities. This would be mechanistically plausible, since syndecan-4 shedding in response to TNF-alpha is mediated by matrix metalloproteinase 9 (Ramnath et al., 2014), and Pycnogenol® intake has been reported to inhibit MMP-9 secretion (Grimm et al., 2006a; Weichmann and Rohdewald, 2024) To the best of our knowledge, a direct inhibition of syndecan-4 and hyaluronan shedding by physiological serum concentrations of polyphenols has not been described previously.

In the present study consistent changes were observed across all assays indicating that serum obtained following Pycnogenol® supplementation positively modulated glycocalyx-associated endothelial properties, with particularly pronounced protective effects against TNF-alpha induced inflammatory injury. Impairment of GCX structures and increased endothelial permeability have been attributed to various factors such as oxidative stress or activation of matrix metalloproteases (MMPs) (Alexander and Elrod, 2002; Becker et al., 2015; Tarbell and Cancel, 2016; Alvarez et al., 2024). The results of the present study are consistent with previous investigations showing that the intake of Pycnogenol® increased antioxidant capacity and downregulated inflammatory processes, e.g., via decrease of NF-κB activation and inhibition of MMP release and activity (Weichmann and Rohdewald, 2024).

## 5 Limitations

Despite the consistent results obtained under dynamic culture conditions, the model system has several limitations. Dynamic cultivation was performed using a shaker and does not represent a defined, directed shear stress, such as generated in physiological flow models with perfusion chambers. The mechanical stress actually applied to the cells cannot be precisely quantified in this experimental setup. However, a shaker represents an established and accessible approach to expose endothelial cells to shear stress (Velasco et al., 2016; Warboys et al., 2019) and it reproducibly promoted the formation of glycocalyx-associated structures, making it a suitable model for studying these structures under mechanical stimulation. Future studies should further investigate the influence of Pycnogenol® under controlled flow conditions with defined shear stress. WGA staining allows conclusions about glycosylated surface structures of the cells but does not provide a direct structural image of the GCX, such as its thickness or exact composition, which require methods like atomic force microscopy. However, the specificity of the WGA signal for glycosylated structures was confirmed by N-acetyl-D-glucosamine blocking, and in combination with the functional permeability measurements and the quantification of shedding markers, the staining contributed to a coherent overall assessment of glycocalyx-associated structures. Furthermore, the present study neither investigated the specific molecular mechanisms underlying the observed effects nor allowed for the attribution of effects to single components or metabolites. While constitutive serum matrix components, such as albumin or other plasma proteins, are unlikely to account for the before/after differences given the paired design, changes specifically induced by the 1-week intervention period but not necessarily attributable to Pycnogenol®-derived polyphenols, such as changes in circulating extracellular vesicles, cannot be excluded as contributing mediators (Jimenez-Trinidad et al., 2025). Notably, the use of serum samples collected after intake of Pycnogenol® reflected physiologic concentrations of all bioavailable metabolites that reach the endothelium *in vivo* (Bayer and Högger, 2024). Further investigations should elucidate precise effect mechanisms and the panel of compounds contributing to the mechanism of action. Serum from the six donors was pooled to investigate a general effect rather than donor-specific responses; the available serum volumes would not have been sufficient for the full assay panel tested for each donor separately. Consequently, the values reflect technical replication of pooled specimen rather than independent biological replicates. The serum experiments should therefore be regarded as hypothesis-generating rather than evidence of population-level efficacy. Additionally, the pre-post design, including the 48-h polyphenol-restricted washout preceding baseline, cannot exclude temporal or order effects or regression to the mean; a placebo-controlled design would be needed to formally attribute the effects to Pycnogenol® intake.

## 6 Conclusion

In summary, this study demonstrated that dynamic culture conditions using a shaker promoted the formation of more pronounced glycocalyx-associated surface structures in human endothelial cells compared to static culture. This was reflected in increased WGA signal intensity per cell, enhanced functional activity of the endothelial monolayer, and greater functional responsiveness to pathophysiological stimuli. This suggests that the dynamic culture model is a suitable *in vitro* model for investigating the biology of the endothelial GCX.

The dynamic cell culture model was subsequently successfully used for the investigation of the effects of serum samples obtained from healthy participants before and after intake of Pycnogenol® on endothelial GCX properties. The consistent and significant increases in WGA signal intensity per cell, the reduction in FITC-dextran permeability, and lower concentrations of the GCX shedding markers syndecan-4 and hyaluronan under all experimental conditions suggested that bioavailable and bioactive Pycnogenol® constituents and/or metabolites may have contributed to stabilizing the endothelial GCX and improved barrier integrity. This protective effect was most pronounced under TNF-alpha mediated inflammatory conditions, suggesting that Pycnogenol®-derived serum components might attenuate inflammatory endothelial damage.

These findings suggest the potential of Pycnogenol® for supporting the integrity of the endothelial GCX and provide an explanation for previously described improvement of vascular health (Weichmann and Rohdewald, 2024). However, this should be verified in studies using individual donor samples and a controlled study design.

## Data Availability

The original contributions presented in the study are included in the article/Supplementary Material, further inquiries can be directed to the corresponding author.
