# Supplementary material for: Influence of dietary supplementation with French maritime pine bark extract (Pycnogenol®) on endothelial glycocalyx structures in a dynamic cell culture model
Source: Front Pharmacol. 2026 Oct 8;17:1949994. doi: 10.3389/fphar.2026.1949994 (PMC13687319; doi:10.3389/fphar.2026.1949994)
Supplement: Supplementary file 1 [file Table1.docx]

Supplementary Material

# 1 Supplementary Table S1: Individual absorbance values of normalized CCK-8 signal obtained from five independent experiments under static and dynamic conditions

| Condition | N1 | | N2 | | N3 | | N4 | N5 | | |
| --- | --- | --- | --- | --- | --- | --- | --- | --- | --- | --- |
| Static:  Control  1 h NaCl  16 h TNF-alpha | 0.003996098  0.003300993  0.004560965 | 0.003789126  0.004226950  0.005549367 | | 0.005600201  0.007249265  0.007220000 | | 0.005598936  0.005834744  0.006076064 | | | 0.005236816  0.006353977  0.007767695 |  |
| Dynamic:  Control  1 h NaCl  16 h TNF-alpha | 0.005010497  0.005829932  0.004906094 | 0.006452301  0.007187736  0.006516978 | | 0.008821379  0.007599693  0.009081475 | | 0.007346406  0.009071898  0.007694504 | | | 0.009601550  0.008280000  0.010379124 |  |

# 2 Supplementary Table S2: Individual values of normalized WGA fluorescence intensity obtained from five independent experiments

| Condition | N1 | | N2 | | N3 | | N4 | N5 | |
| --- | --- | --- | --- | --- | --- | --- | --- | --- | --- |
| Static:  Control  1 h NaCl  16 h TNF-alpha | 26131.18  21264.81  19960.93 | 23653.98  23810.83  20914.31 | | 23466.88  26961.82  26117.34 | | 26018.30  26696.90  24898.53 | | | 25272.16  22372.57  27359.83 |
| Dynamic:  Control  1 h NaCl  16 h TNF-alpha | 32911.71  29899.20  21352.61 | 39033.86  27996.90  21838.25 | | 36422.81  27818.69  25639.42 | | 41825.51  27785.91  33575.58 | | | 47444.89  36201.30  31558.67 |
| Before Intake:  Control  1 h NaCl  16 h TNF-alpha | 65013.80  59399.59  56996.26 | 77283.09  66471.18  62870.39 | | 76103.64  68417.20  58314.51 | | 75208.12  67691.69  68120.09 | | | 79294.33  69907.61  65335.95 |
| After Intake:  Control  1 h NaCl  16 h TNF-alpha | 94501.86  83527.04  88151.02 | 100294.27  89388.86  84989.86 | | 121581.62  102241.70  107305.47 | | 97174.01  89787.18  92915.23 | | | 119072.69  113376.12  113272.96 |

# 3 Supplementary Table S3: Results of the WGA-signal blocking assay: Data are presented as mean ± SEM. Values obtained under static and dynamic culture conditions are based on five independent experiment (n = 5), whereas values obtained for the serum experiments are based on three independent experiment (n = 3). All blocked conditions differed significantly from corresponding unblocked control (< 0.05)

| Condition | Static | Dynamic | Before Intake | After Intake |
| --- | --- | --- | --- | --- |
| Control | 4820 ± 117 | 4935 ± 504 | 15813 ± 1248 | 15489 ± 216 |
| 1 h NaCl | 5012 ± 164 | 6311 ± 326 | 14345 ± 168 | 14669 ± 195 |
| 16 h TNF-alpha | 5344 ± 506 | 5082 ± 760 | 13633 ± 804 | 15419 ± 663 |

# 4 Supplementary Table S4: FITC-dextran permeability assay under static and dynamic culture conditions. Data are presented as mean ± SEM from five independent experiment (n = 5). No significant differences were observed between static and dynamic culture conditions or following stimulation with NaCl or TNF-alpha. The mean permeability coefficient (P_app) of a cell-free (blank) transwell insert, measured in parallel with each independent experiment, was 26.84 ± 1.38 ×10⁻⁶ cm/s (n = 5).

| Condition | Static  FITC-dextran permeability (%) | Dynamic  FITC-dextran permeability (%) | Static  P_app (*10^-6^ cm/s) | Dynamic  P_app (*10^-6^ cm/s) |
| --- | --- | --- | --- | --- |
| Control | 33.26 ± 1.62 | 32.83 ± 2.28 | 9.40 ± 4.40 | 8.72 ± 3.94 |
| 1 h NaCl | 33.23 ± 1.78 | 33.34 ± 2.10 | 9.37 ^±^ 4.40 | 9.26 ± 4.39 |
| 16 h TNF-alpha | 34.71 ± 1.22 | 33.94 ± 1.69 | 10.18 ± 4.80 | 9.53 ± 4.47 |

# 5 Supplementary Table S5: Individual values of syndecan-4 and hyaluronan concentrations (pg/10^5^ cells) obtained from five independent experiments under static and dynamic conditions.

| Condition | Static  Syndecan-4 | Dynamic  Syndecan-4 | Static  Hyaluronan | Dynamic  Hyaluronan |
| --- | --- | --- | --- | --- |
| N1  Control  1 h NaCl  16 h TNF-alpha | 133.97  136.37  140.77 | 132.98  125.65  140.04 | 42.13  44.77  51.23 | 45.39  51.23  59.62 |
| N2  Control  1 h NaCl  16 h TNF-alpha | 115.68  110.09  122.83 | 137.94  123.51  134.10 | 43.09  38.67  50.78 | 58.50  55.56  65.05 |
| N3  Control  1 h NaCl  16 h TNF-alpha | 106.97  114.74  113.22 | 124.63  125.45  133.23 | 48.73  52.97  49.62 | 69.46  61.05  55.7 |
| N4  Control  1 h NaCl  16 h TNF-alpha | 107.44  113.22  119.43 | 127.85  130.71  132.14 | 51.94  54.54  44.41 | 69.82  56.62  59.48 |
| N5  Control  1 h NaCl  16 h TNF-alpha | 108.92  114.49  123.69 | 125.81  131.02  142.05 | 50.87  54.78  62.34 | 60.03  67.69  80.50 |

*.*

# 6 Supplementary Table S6 : Normalized CCK-8 absorbance values under dynamic cell culture conditions when cells were cultured with serum samples from human volunteers obtained before and after supplementation with Pycnogenol^®^: Data are presented as mean ± SEM from three independent experiments (n = 3). ^†^ Comparison between before and after conditions within the respective treatment group (two-way ANOVA followed by Tukey post-hoc test) ^*^ p < 0.05 compared with the respective untreated control (two-way ANOVA followed by Tukey post-hoc test)

| Condition | Before Intake | After Intake | p-value^†^ |
| --- | --- | --- | --- |
| Control | 0.003184 ± 0.000254 | 0.003026 ± 0.000301 | 0.1508 |
| 1 h NaCl | 0.003390 ± 0.000375 | 0.003504 ± 0.000358 | 0.3190 |
| 16 h TNF-alpha | 0.003312 ± 0.000229 | 0.003420 ± 0.000352 | 0.6932 |

# 7 Supplementary Table S7: Individual values of FITC-dextran permeability (%) and P_app (*10^-6^ cm/s) obtained from five independent experiments under dynamic cell culture conditions when cells were cultured with serum samples from human volunteers obtained before and after supplementation with Pycnogenol^®^

| Condition | Before Intake  FITC-dextran permeability (%) | After Intake  FITC-dextran permeability (%) | Before Intake  P_app (*10^-6^ cm/s) | After Intake  P_app (*10^-6^ cm/s) |
| --- | --- | --- | --- | --- |
| N1  Control  1 h NaCl  16 h TNF-alpha | 25.66  31.22  37.51 | 14.35  17.46  18.69 | 1.34  1.63  1.96 | 0.75  0.91  0.98 |
| N2  Control  1 h NaCl  16 h TNF-alpha | 29.54  35.06  40.10 | 17.28  20.76  21.25 | 3.06  3.64  4.16 | 1.71  2.15  2.20 |
| N3  Control  1 h NaCl  16 h TNF-alpha | 27.73  34.07  39.56 | 16.65  19.97  20.93 | 1.51  1.85  2.15 | 0.90  1.09  1.14 |
| N4  Control  1 h NaCl  16 h TNF-alpha | 32.30  38.64  46.57 | 17.31  21.40  22.14 | 1.60  1.92  2.31 | 0.86  1.06  1.10 |
| N5  Control  1 h NaCl  16 h TNF-alpha | 28.70  34.82  41.62 | 16.33  20.15  21.01 | 1.33  1.61  1.92 | 0.76  0.93  0.97 |

# 8 Supplementary Table S8: Individual values of syndecan-4 and hyaluronan concentrations (pg/10^5^ cells) obtained from five independent experiments under dynamic cell culture conditions when cells were cultured with serum samples from human volunteers obtained before and after supplementation with Pycnogenol^®^

| Condition | Before Intake  Syndecan-4 | After Intake  Syndecan-4 | Before Intake  Hyaluronan | After Intake  Hyaluronan |
| --- | --- | --- | --- | --- |
| N1  Control  1 h NaCl  16 h TNF-alpha | 29.57  27.06  37.51 | 25.00  22.54  28.93 | 57.29  61.14  74.50 | 45.29  47.95  49.90 |
| N2  Control  1 h NaCl  16 h TNF-alpha | 41.58  37.42  45.96 | 28.00  25.30  31.43 | 85.38  94.33  99.12 | 66.47  68.11  66.14 |
| N3  Control  1 h NaCl  16 h TNF-alpha | 43.37  37.23  52.45 | 28.25  24.66  29.34 | 73.25  84.86  86.10 | 50.36  53.65  59.54 |
| N4  Control  1 h NaCl  16 h TNF-alpha | 49.06  48.70  60.72 | 27.51  25.93  30.59 | 69.02  76.06  91.54 | 44.88  50.10  56.31 |
| N5  Control  1 h NaCl  16 h TNF-alpha | 31.95  33.12  35.73 | 26.80  28.82  28.34 | 68.37  66.53  76.34 | 44.41  56.39  60.69 |
